# Green synthesis of copper oxide nanoparticles using walnut shell and their size dependent anticancer effects on breast and colorectal cancer cell lines

**DOI:** 10.1038/s41598-024-71234-4

**Published:** 2024-09-02

**Authors:** Hanieh Abdollahzadeh, Yaghub Pazhang, Asghar Zamani, Yousef Sharafi

**Affiliations:** 1 Department of Biology, Faculty of Sciences, Urmia University, Urmia, Iran; 2 Department of Cellular and Molecular Biotechnology, Institute of Biotechnology, Urmia University, Urmia, Iran; 3 Department of Nanotechnology, Faculty of Chemistry, Urmia University, Urmia, Iran; 4 Dryland Agricultural Research Institute, Agricultural Research, Education and Extension Organization(AREEO), Maragheh, Iran

**Keywords:** Walnut shell, Green synthesis, CuO nanoparticles, Cancer therapy, Apoptosis, p53, Nrf-2, Biochemistry, Cancer, Cell biology, Chemical biology, Neuroscience, Plant sciences, Nanoscience and technology

## Abstract

Metal oxide nanoparticles(NPs) contain unique properties which have made them attractive agents in cancer treatment. The CuO nanoparticles were green synthesized using walnut shell powder in different calcination temperatures (400°, 500°, 700°, and 900 °C). The CuO nanoparticles are characterized by FTIR, XRD, BET, SEM and DLS analyses. SEM and DLS analyses showed that by increasing the required calcination temperature for synthesizing the NPs, their size was increased. DPPH analysis displayed no significant anti-oxidative properties of the CuO NPs. The MTT analysis showed that all synthesized CuO NPs exhibited cytotoxic effects on MCF-7, HCT-116, and HEK-293 cell lines. Among the CuO NPs, the CuO-900 NPs showed the least cytotoxic effect on the HEK-293 cell line (IC_50_ = 330.8 µg/ml). Hoechst staining and real-time analysis suggested that the CuO-900 NPs induced apoptosis by elevation of p53 and Bax genes expression levels. Also, the CuO-900 NPs increased the Nrf-2 gene expression level in MCF-7 cells, despite the HCT-116 cells. As can be concluded from the results, the CuO-900 NPs exerted promising cytotoxic effects on breast and colon cancer cells.

## Introduction

Metal oxide nanoparticles have gained more attentions rather than other nanoparticles due to their unique properties and their applications in various fields, such as anti-microbial agents, in semi-conducting devices, textile industry, microelectronics, cosmeceutical products, and biomedical fields^1^. Because of the emerging anticancer effects of the metal oxide NPs, they have been considered as agents to remove cancer cells in preclinical studies^2^. In recent years, the synthesis of CuO nanoparticles has been the goal of scientific research because of their various application especially biomedical applications^3–5^. It has been reported that CuO nanoparticles are more cytotoxic to human cells rather than other metal oxide NPs^6^. However, the anti-cancer effects of CuO NPs were studied on many cancer types, including liver^7^, lung^8^, and breast^9,10^, cervical^11^ and pancreatic^12^ cancers. In 2023, 1,958,310 new cancer cases and 609,820 cancer deaths are estimated to occur in the United States^13^. Despite the existence of multiple approaches to treat cancer including chemotherapy, immunotherapy, radiotherapy, and surgery, not only the disease is still a main health challenge in worldwide but also its related deaths are increasing year by year^13^. Therefore, finding new agents and approaches to treat cancer is one of significant health challenges in the field of medical science. Based on the WHO report, three prevalent cancer types in women include breast, lung and colorectal cancers. On another hand, the most prevalent cancer types in the men are prostate, lung, and colorectal cancers^14^. Increase in cancer prevalence is related partly to the resistance of cancer cells to the conventional therapies. Beside the conventional therapeutic strategies, capabilities of nanoparticles as an agent to treat cancer and to effectively deliver anticancer drugs have made them as a hopeful approach for overcoming cancer^15^.

Numerous synthesis procedures have been developed to synthesize CuO nanoparticles, including co-precipitation, sol–gel technique, thermal decomposition, and hydrothermal methods. However, these techniques, in addition to the environmental impacts, are usually hard to produce on the industrial scale. Accordingly, more attention has been recently paid to the green approach and biological method to the synthesis of nanoparticles, chiefly copper oxide nanoparticles^16–19^.

Agricultural waste biomass particularly lignocellulosic wastes such as nut shells have increasingly attracted some attention as a low-cost renewable resource in various fields^20,21^. Walnut is always a popular fruit in the world. Based on incomplete statistics, thousands of tons of walnuts are consumed every year in the world and the shells are usually cast-off. More recently, we synthesized nanostructured magnesium oxide^22^, alumina^23^, and cerium oxide nanoparticles^24^ by walnut shell. This abundantly available agricultural waste is composed of cellulose, hemicellulose, and lignin^5,25^ which can act as fuel or sacrificial templates in preparing metal oxide nanostructures. In this protocol, walnut shell grind was mixed with aqueous solutions of metal nitrate in diverse ratios without using any toxic chemicals. Stirring followed by evaporation and calcination of paste, resulting in desired nanostructures. In this paper, we present similar process for the preparation of CuO nanoparticles with different sizes by using walnut shell powder. The aim of this study is the green synthesis of different dimensions of CuO NPs to investigate their anticancer effects.

## Material and methods

### General remarks

Copper(II) nitrate hexahydrate, Cu(NO_3_)_2_.6H_2_O, from Merck and used without further purification. The walnut shell from a local walnut tree in Urmia (Iran) was crushed using a high-speed rotary cutting mill. X-ray diffraction patterns of the obtained materials were recorded at room temperature on Shimadzu XRD-6000 diffractometer with CuKα irradiation. The morphology of materials was observed by Hitachi S-4100 FESEM instrument (Japan). Also, BET analysis was performed by Belsorp-mini II-BEL, Inc. analyzer at 77 K. Fourier transform infrared (FT-IR) spectra were attained by a Bruker Vector 22 FT-IR spectrophotometer under ambient conditions in a KBr/Nujol mull in the range of 400–4000 cm^−1^.

### Copper oxide nanoparticles preparation

**Fig. 1:**
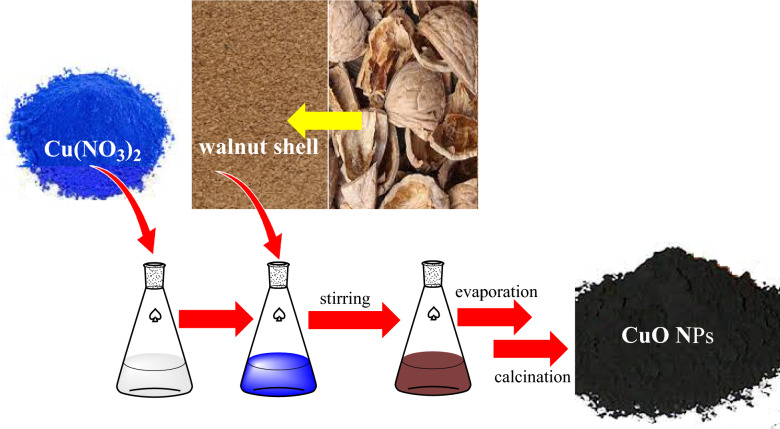
Schematic diagram of copper oxide NP preparation.

Walnuts were purchased from local farmers and then the walnut shells separated from the kernel. Next, the walnut shells were grinded and the walnut shell powder was prepared. Copper oxide NPs was synthesized in the presence of walnut shell powder 30 g and copper(II) nitrate hexahydrate 6.65 g in 50 mL of deionized water (Millipore, Milli-Q grade). After 4 h stirring, the water was evaporated by a rotary evaporator under low pressure. The resulting paste was subsequently calcined at 400 °C for 4 h (at a heating rate of 10 °C/min) under open-air conditions to give a black solid (CuO-400). For comparison, CuO-500, CuO-700, and CuO-900 NPs were prepared at 500, 700, and 900 °C, respectively. A schematic diagram of Copper oxide NP preparation is shown in Fig. 1.

### CuO NPs DPPH radical scavenging capacity.

DPPH test was employed to measure the CuO NPs antioxidant capacity. Shortly, the CuO NPs added to 3 ml of 0.1 mM of DPPH (Sigma) methanolic solution (Merck) at different concentrations (12.5 to 400 µg/ml) and then kept in dark at 37 °C for 30 min. The methanolic solution was applied as a control, and the Butylated hydroxyl anisole (BHA, Sigma) solution was used as standard. Next, the samples absorbance was read at 517 nm by a spectrophotometer. Eventually, the CuONPs free radical scavenging activity was evaluated by Eq. (1):

1 $$\% {\text{ Free Radical Scavenging Capacity}} = \left( {\left( {{\text{Ac}} - {\text{As}}} \right)/{\text{Ac}}} \right) \, \times {1}00$$

In Eq. (1), the Ac and As are OD of control and standard or CuO NPs in methanolic solution, respectively^26^.

### Cell culture and treatment

HCT-116, MCF-7 (cancer cells) and HEK-293 cells (healthy or normal cell) were obtained from the iranian pasture institute. Then, the cells maintained in DMEM high glucose medium (Gibco) containing 10% FBS (Gibco) and glutamine (Merck) enricnhed with 1% penicillin–streptomycin(Biowest) in CO_2_-incubator at 37 °C. For evaluation the cytotoxic effect of the CuO nanoparticles produced at different temperatures (400 °C, 500 °C, 700 °C, and 900 °C) as well as CuO bulk, 5 × 10^3^ cells were seeded in 96 well plate and exposed to different concentrations of the nanoparticles(20,40, 80 µg/ml prepared in distilled water) for 72 h at 37 °C. After that, 10 µl of MTT dye solution (5mg/ml, Sigma) was added to each well and the plates were incubated at 37 °C in dark. Next, after removing each well medium, 100µl DMSO (Dimethyl sulfoxide, Merck) was poured into each well to dissolve the formazan crystals. Finally, the wells absorbance was read at 570 nm and based on the control wells absorbance, the treated cells viability were calculated and the plots drawn by GraphPad prism Ver. 9.0.0.

### Hoechst staining to monitor apoptosis

In the apoptosis process, cells undergo some morphological alterations including nucleus condensation and fragmentation as well as apoptotic body formation^27^. The Hoechst33342 dye can bind to DNA and monitor the cell nucleus shape. First 2 × 10^5^cells(HCT-116 and MCF-7 cell line) were seeded in each well of 24 well plate, then treated with 80µg/ml concentrations of the CuO nanoparticles produced at 400, 500, 700, and 900 °C as well as CuO bulk for 72 h. In order to Hoechst staining, the cells first washed once by PBS(phosphate buffered saline)and trysinized that followed by fixing in ice cold methanol(Merck) at − 20 °C for 30 min. Thereafter, the cells were centrifuged and the supernatant was discarded and the cell pellets were twice washed in PBS. In the next step, the cells stained by Hoechst 33,342 satin solution(Sigma, 1mg/ml) for 30 min out of light. Finally, the cells were transferred on glass coverslips and photographed by a fluorescent microscope (Zeiss) by a DAPI filter.

### Q-PCR to analyze apoptotic and antioxidant genes expression

To evaluate the apoptotic and antioxidant genes expression by real-time PCR method, first, 2 × 10^6^ MCF-7 and HCT-116 cells were seeded in each well of 6-well plate and then exposed to 80 µg/ml concentration of CuO-900 NPs. After that, the cells were trypsinized and centrifuged to obtain the cells pellets. Next, the cells RNAs were extracted by RNXplus kit(Sinaclon, Iran) based on manufacture’s protocol. After determining the RNAs concentrations by a Nanodrop (Biotek), the cDNA synthesis was performed using 1000 ng extracted RNA by cDNA synthesis kit(Parstus, Iran). Next, the samples cDNA were subjected to real-time PCR (Applied Biosystems) in the presence of Bax, Bcl-2, p53, Nrf-2, superoxide dismutase, catalase and Beta-action(as loading control) primers using sybergreen mastermix(Ampliqon) as the following steps:

Denaturation at 94 °C for 5 min, 40 cycles of Denaturation at 94 °C for 30 s, annealing at 58 °C for 30 s, elongation at 72 °C for 30 s.

All the primers sequence was designed by using Oligo 7 software, the primers sequences have been presented at Table 1.

**Table 1:** primer sequences of apoptotic and antioxidant genes.

Gene	Primer	Primer sequence 5ʹ-3ʹ
Bax	Forward	CGAGCAGGGCGAATGGGGG
	Reverse	GTCCCCGATGCGCTTGAGA
Bcl-2	Forward	GGGGTGAACTGGGGGAGGATTG
	Reverse	TGCCGGTTCAGGTACTCAGTCA
P53	Forward	CAACTCACTAGGGGAACCAAAC
	Reverse	AATGCGGACTCTGAACTGATGC
Nrf-2	Forward	AGAGCTAGATAGTGCCCCTGGA
	Reverse	ATGACCAGGACTTACAGGCAAT
CAT	Forward	AGCTGGTTAATGCAAATGGGGA
	Reverse	TGTGGCAATGGCGTTAAAAAGA
SOD	Forward	AGGATGAAGAGAGGCATGTTGG
	Reverse	GTTTCCCGTCTTTGTACTTTCT
β-Actin	Forward	CAACTGGGACGACATGGAGAAA
	Reverse	GATAGCACAGCCTGGATAGCAA

### Statistical analysis

The experiments were repeated at least three times (n = 3) and the obtained data were analyzed via one or two-way ANOVAs using the Tukey post hoc test by GraphPad prism Ver. 9.0.0 software. The p-value < 0.05, 0.01, 0.001, and 0.0001 were considered as significant difference between the control and treated groups.

## Results and discussion

### CuO NPs were synthesized in different sizes

**Fig. 2:**
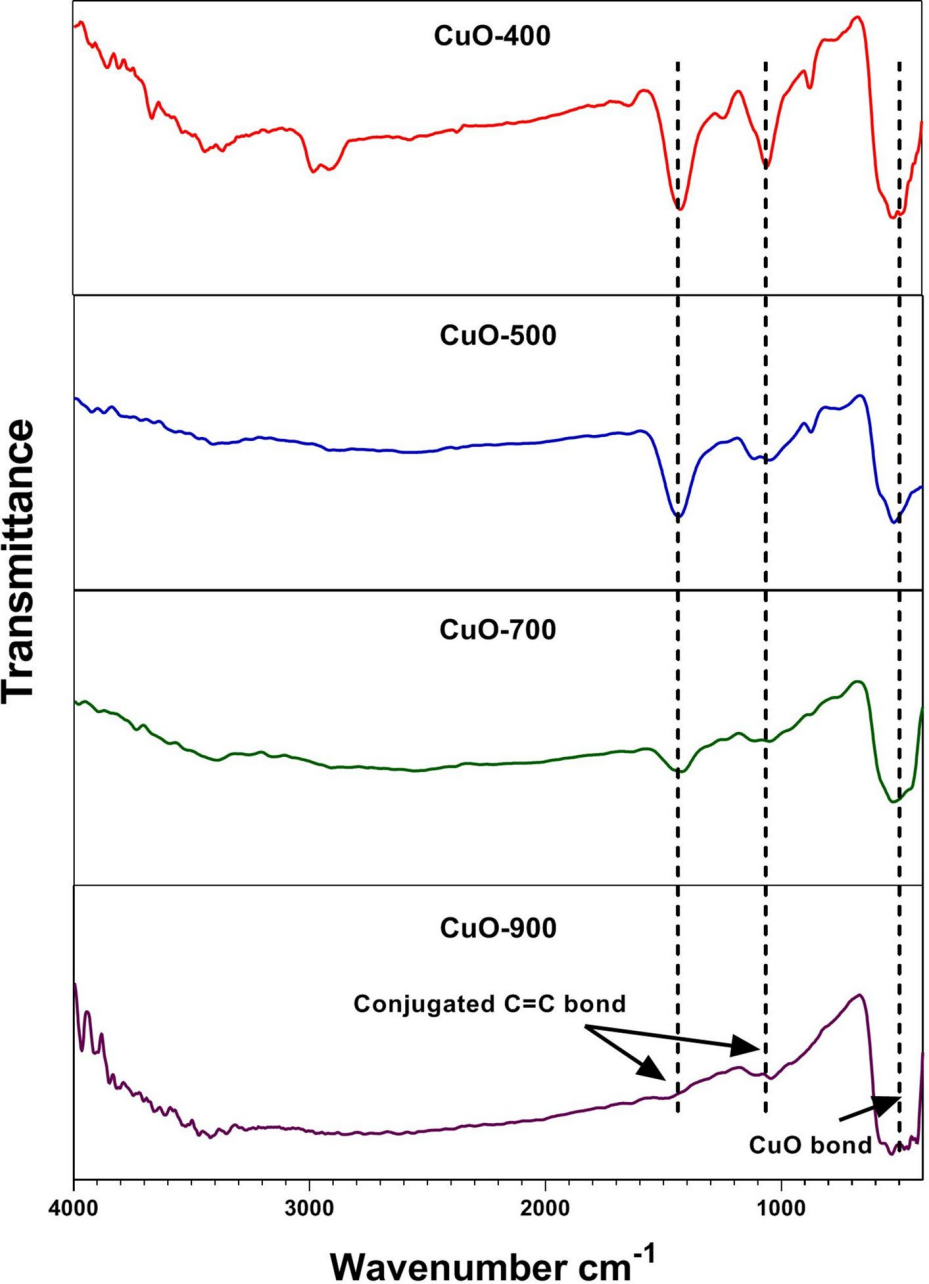
FT-IR analysis. In CuO-900 NPs the peak of wavenumber 1400 cm^−1^ has been disappeared.

The FTIR spectrums of CuO nanoparticles obtained by process described above are presented in Fig. 2. The FTIR spectrum shows peaks at 400–600 cm^-1^, which can be assigned to the vibrations of Cu–O bonds. Also, peaks at around 1400 cm^−1^ are attributed to carbon residue that an increased calcination temperature was followed by removal of carbon impurities and shortening of these peaks.

**Fig. 3:**
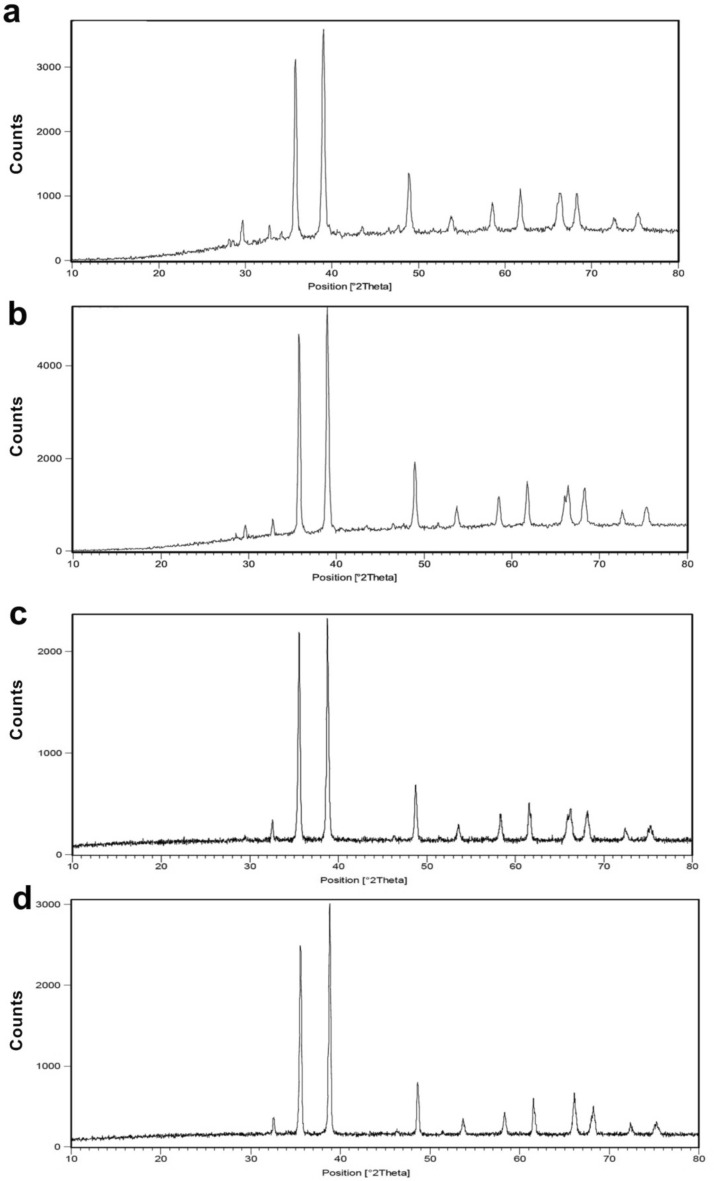
X-ray diffraction (XRD) pattern of different size CuO NPs, (**a**) CuO-400, (**b**) CuO-500, (**c**) CuO-700 and (**d**) CuO-900.

X-ray diffraction(XRD) analysis of synthesized different-sized CuO NPs were shown in Fig. 3. The XRD pattern matched with the monoclinic structure of CuO crystal(JCPDS card 80–1917). An intense diffraction peak at 38.77° was detected corresponding to the lattice plane (111), in addition to this, some low-intensity peaks at 35.74° (002) and 48.94° (202), were also detected. The effect of temperature was also studied on crystal growth, showing the small changes in the sharpness of peaks and growth of (111) and (002) planes in CuO-400, CuO-500, CuO-700, and CuO-900, respectively. The crystallite size of different sizes CuO NPs was determined through Scherrer’s Eq. (2) and full-width half maximum (FWHM) of the most intense (111) peak.

2 $$\text{D }= 0.89\lambda /\beta cos\theta$$

$\lambda$=X-ray wavelength (0.15418 nm), $\beta$= FWHM, D = crystallite size, $\theta =$ Bragg's angle.

The average crystallite size was found to be 28, 32, 35, and 42 nm for CuO-400, CuO-500, CuO-700 and CuO-900, respectively.

Additionally, the specific surface area and particle sizes of CuO NPs can be determined using the BET surface area (Table 1). The method works based on nitrogen gas adsorption at a constant temperature. This analysis indicated the average thermodynamic size of CuO-400 to be 71 nm. It has been observed that with the increase in temperature of calcination the average size increase to 127, 201, and 295 nm for CuO-500, CuO-700, and CuO-900, respectively. An increase in calcination temperature results in growing of crystals (coarsening or Ostwald ripening) and hence higher particle as well as crystallite sizes.

**Fig. 4:**
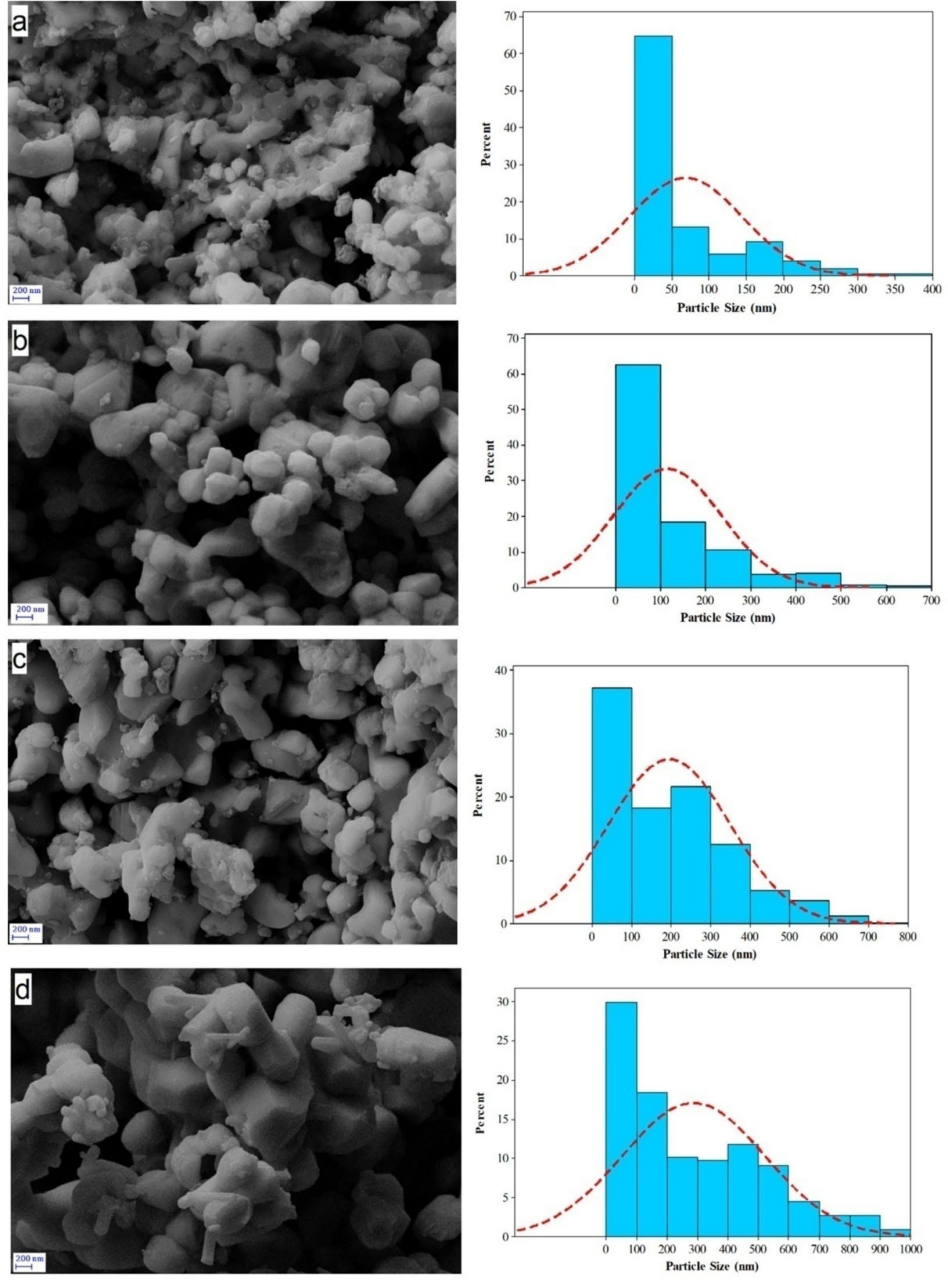
FESEM images and size distribution histogram of (**a**) CuO-400, (**b**) CuO-500, (**c**) CuO-700 and (**d**) CuO-900.

**Fig. 5:**
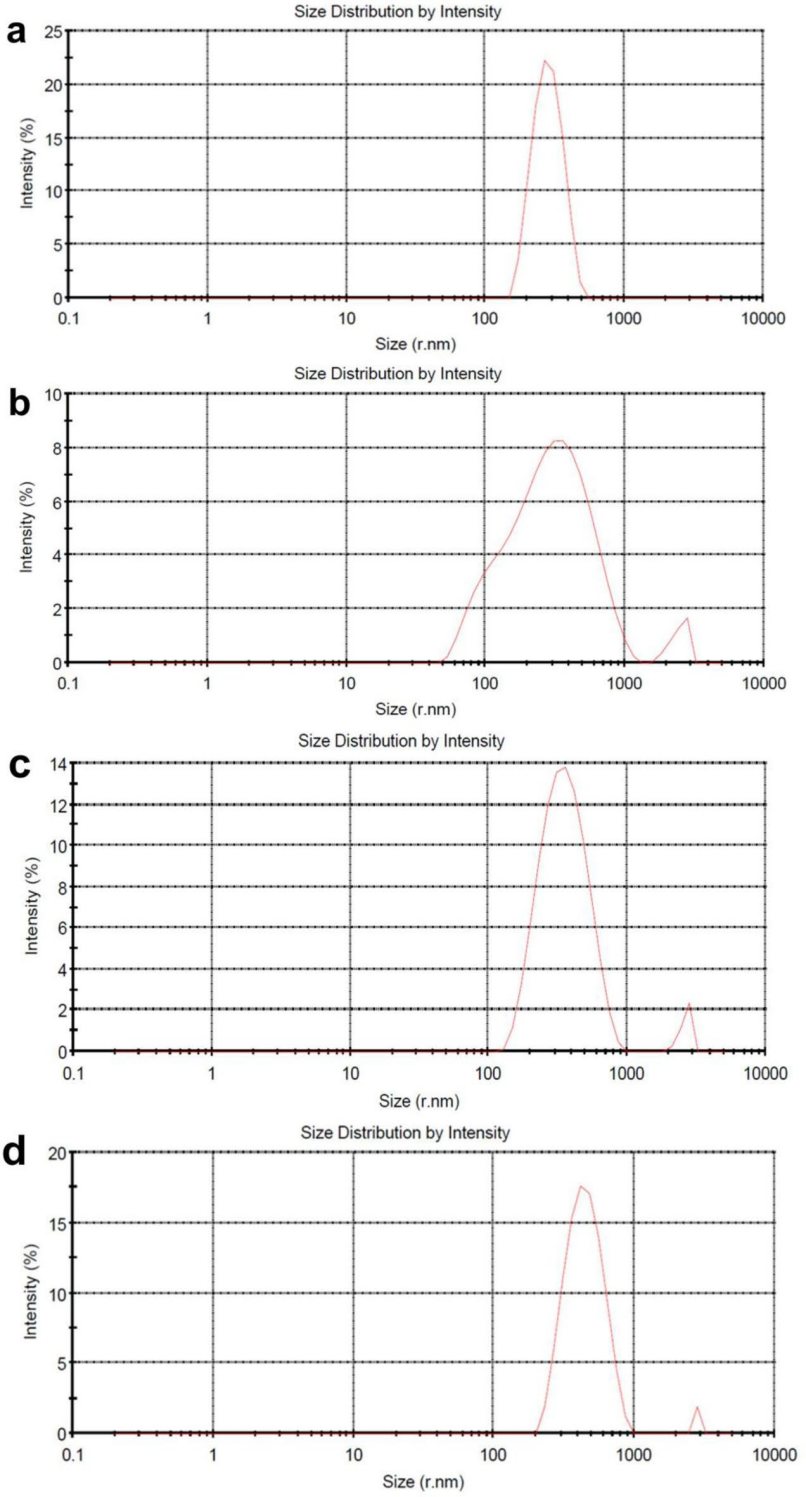
Dynamic Light Scattering analysis of CuO NPs. (**a**) CuO-400, (**b**) CuO-500, (**c**) CuO-700, and (**d**) CuO-900 NPs. As the results show the size of the CuO-NPs were increased by raising the calcinations temperature.

The BET results were further supported by FESEM images and a histogram of the particle size distribution (Fig. 4 and Table 2). From the FESEM images, it is clearly evident that with raising temperature, the average size and size distribution of nanoparticles also increases, and the interesting thing is that rod-like nanoparticles are also seen in CuO-900. It is also evident that nanoparticles at higher temperatures are more geometrically regular than nanoparticles prepared at lower temperatures. By carefully looking at the histograms, it can be seen that the highest frequency of nanoparticles in all 4 samples is related to nanoparticles under 100 nm, and in sample CuO-400, it is related to nanoparticles under 50 nm, but with increasing temperature, the size distribution of nanoparticles has expanded to larger values. As can be seen, the size of the particles obtained from DLS analysis is much larger than the size obtained using the FESEM histogram (Fig. 5). This difference is due to the principle that the DLS technique provides the hydrodynamic diameter of agglomerated particles rather than the real size of nanoparticles^28^.An increase in calcination temperature results in larger size of agglomerated particles and bimodal distribution.

**Table 2:** Surface area, BET particle sizes and FESEM particle sizes of copper oxide nanoparticles.

Sample	BET surface area (m^2^/g)	BET particle size (nm)	FESEM particle sizes (nm)
CuO-400	13.4	71	69
CuO-500	7.5	127	120
CuO-700	4.7	201	195
CuO-900	3.2	295	287

**Fig. 6:**
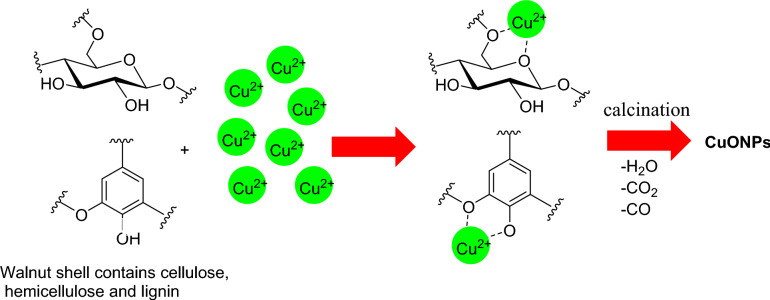
Proposed mechanism of the CuO NPs formation.

The proposed mechanism of the CuO nanoparticle formation is pictured in Fig. 6. It is possible that the Cu^2+^ ions were dispersed on the walnut shell containing cellulose, hemicellulose, and lignin via coordination with their alkoxy, hydroxyl, and other oxygenated groups. Therefore, walnut shell act as an adsorbent for the copper precursors. After adoption and dispersion of the Cu^2+^ on the walnut shell and calcination, the template was removed (walnut shell as sacrified template or fuel) by transformation of CO, CO_2_, and H_2_O (Fig. 6). Simultaneously, due to temperature increase CuO is formed with removal of template. The product should be nanostructured due to two reasons including 1-use of the template and 2- release of gases which increase the surface area (Fig. 6).

**Fig. 7:**
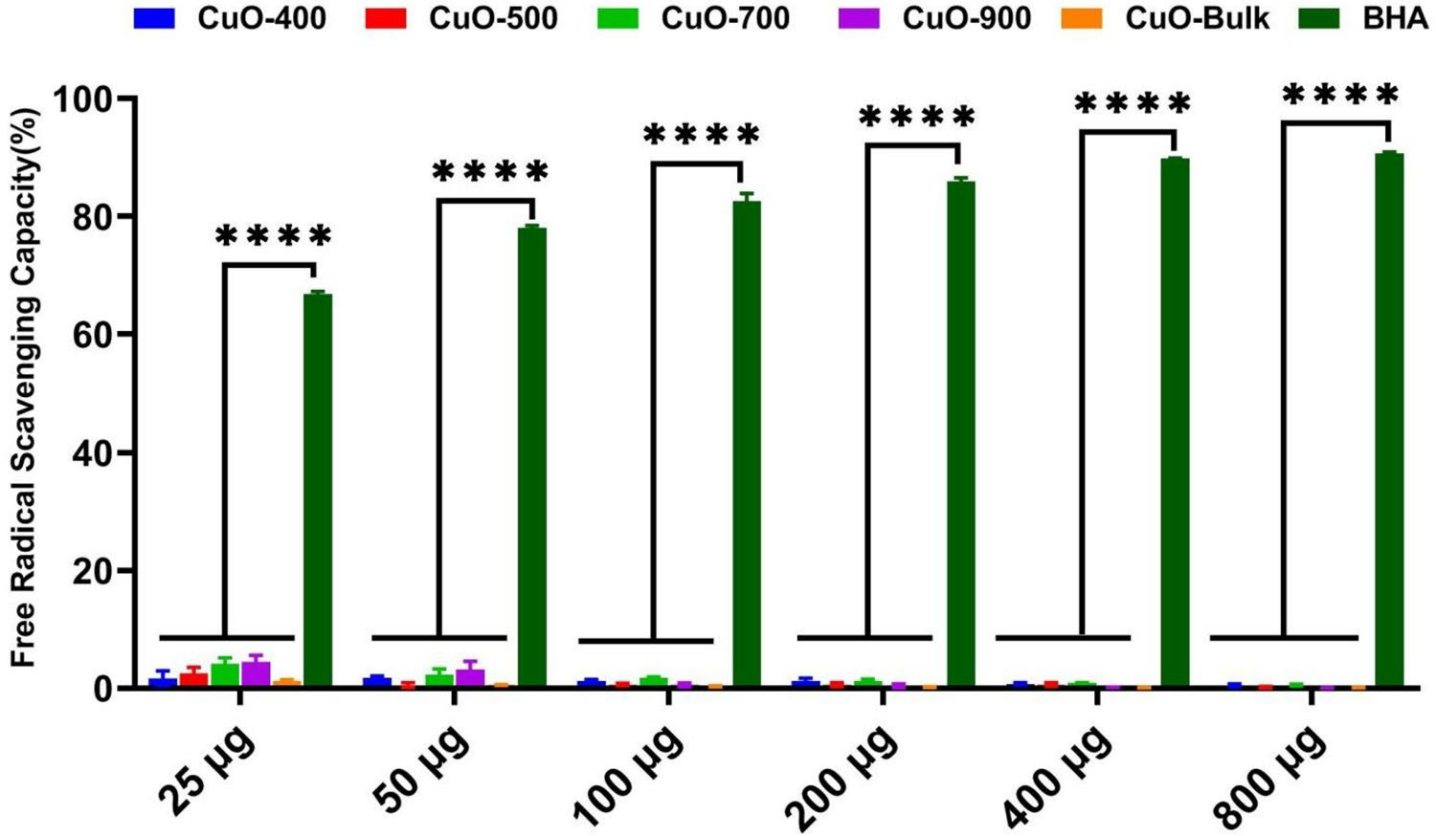
Antioxidant capacity. The CuO NPs showed almost no DPPH scanenging capacity. BHA: Butylated hydroxyl anisole. ****p < 0.0001.

### CuO nanoparticles especially CuO-900 NPs exerted anticancer effects

**Fig. 8:**
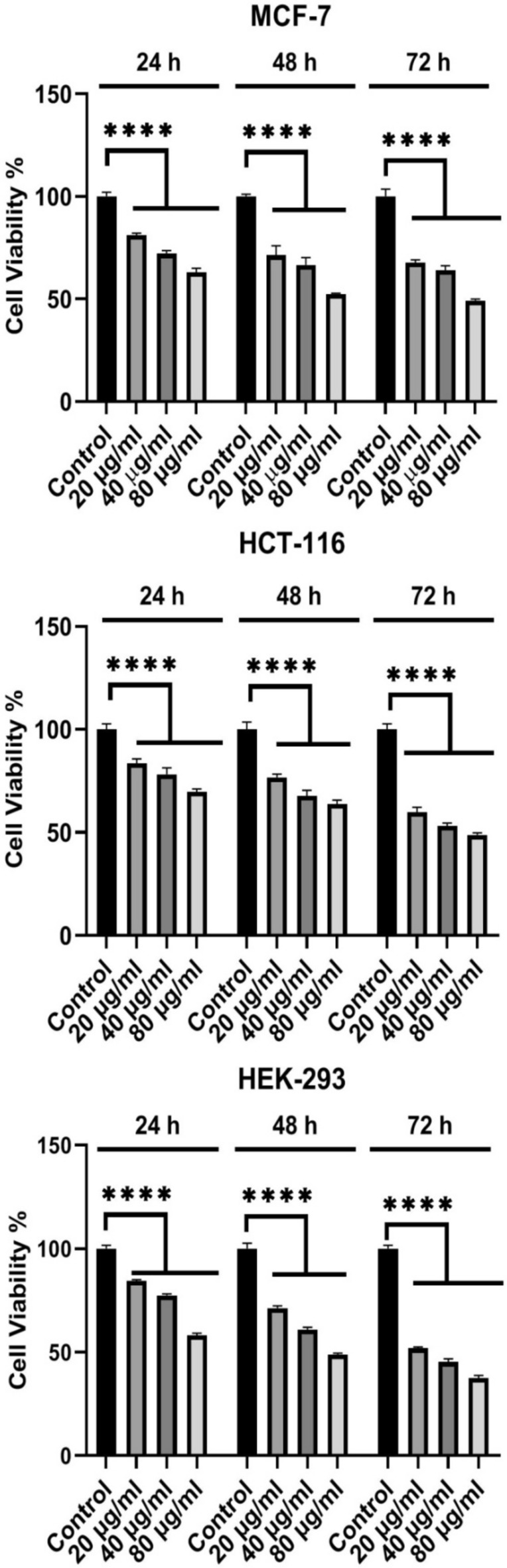
Cytotoxic effects of CuO-400 NPs on MCF-7, HCT-116 and HEK-293 cell lines. CuO-400 NPs exhibited cytotoxic effect in all concentrations. Control: untreated cells. ****p < 0.0001.

**Fig. 9:**
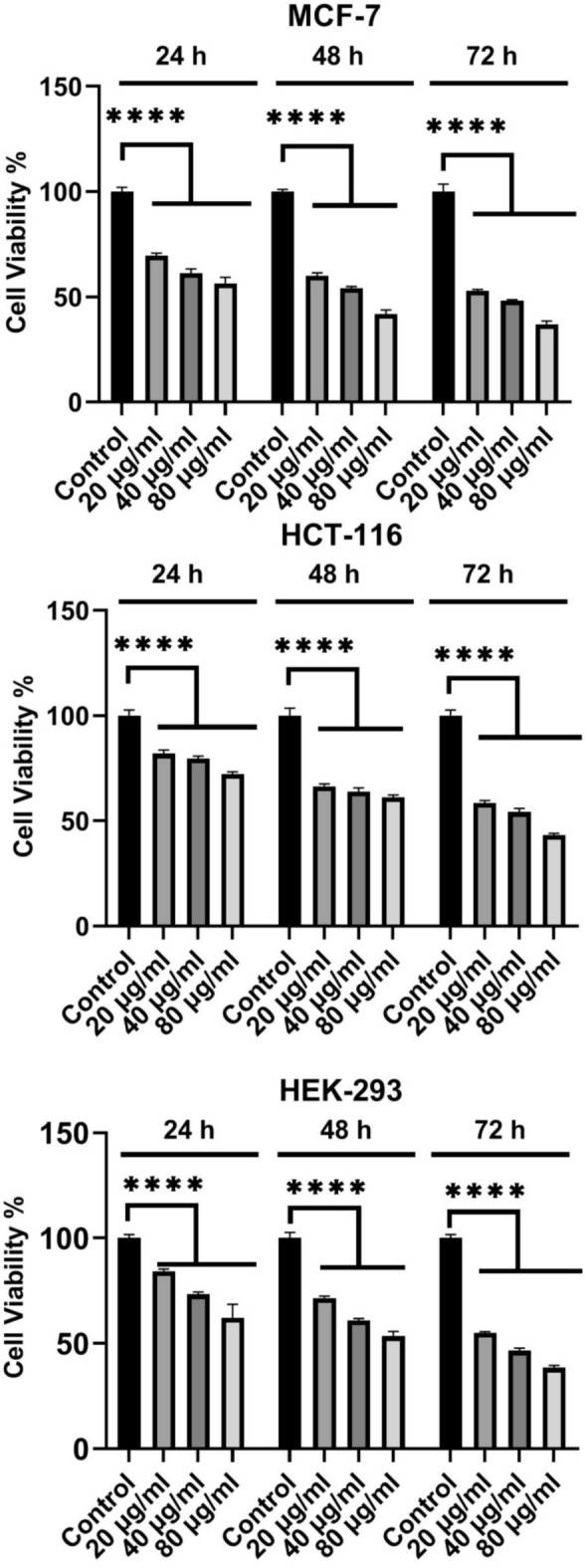
Effect of CuO-500 NPs on MCF-7, HCT-116 and HEK-293 cells viability. CuO-500 NPs reduced viability of the cells. Control: untreated cells. ****p < 0.0001.

**Fig. 10:**
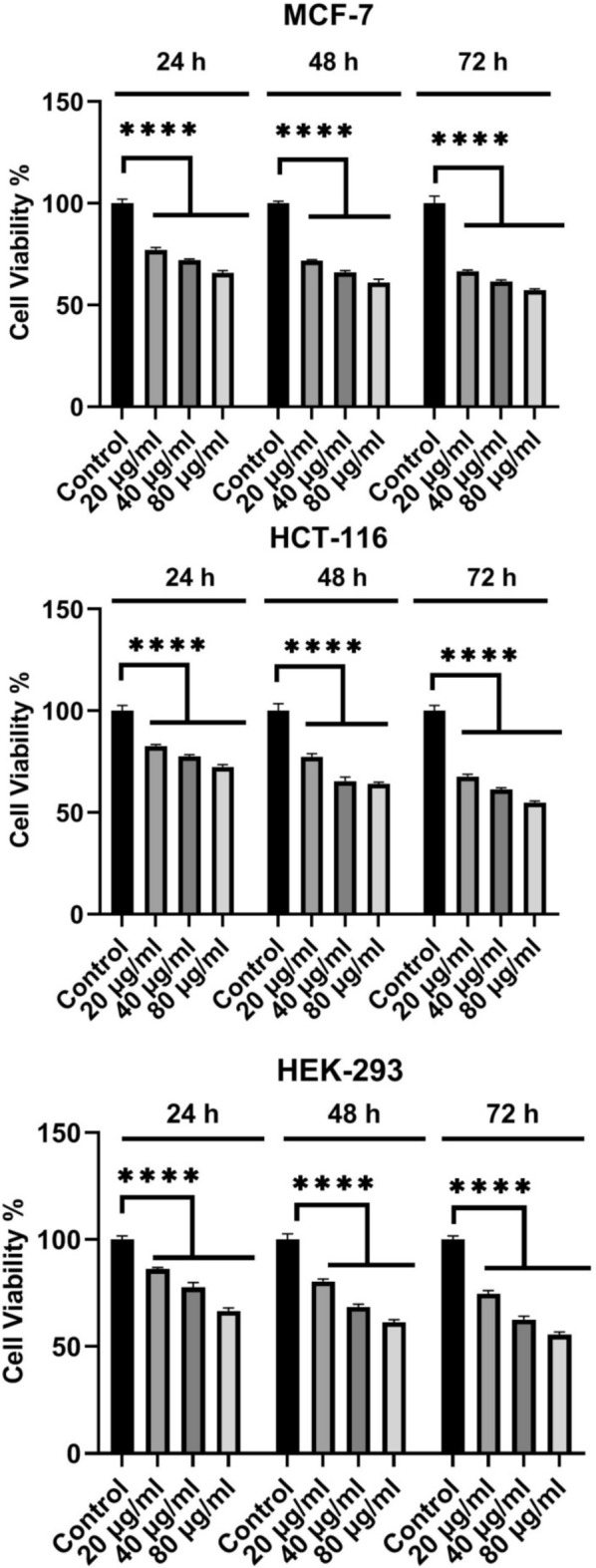
CuO-700 NPs’ cell viability reducing effect. CuO-700 decreased viability of MCF-7, HCT-116 and HEK-293 cell lines. Control: untreated cells. ****p < 0.0001.

**Fig. 11:**
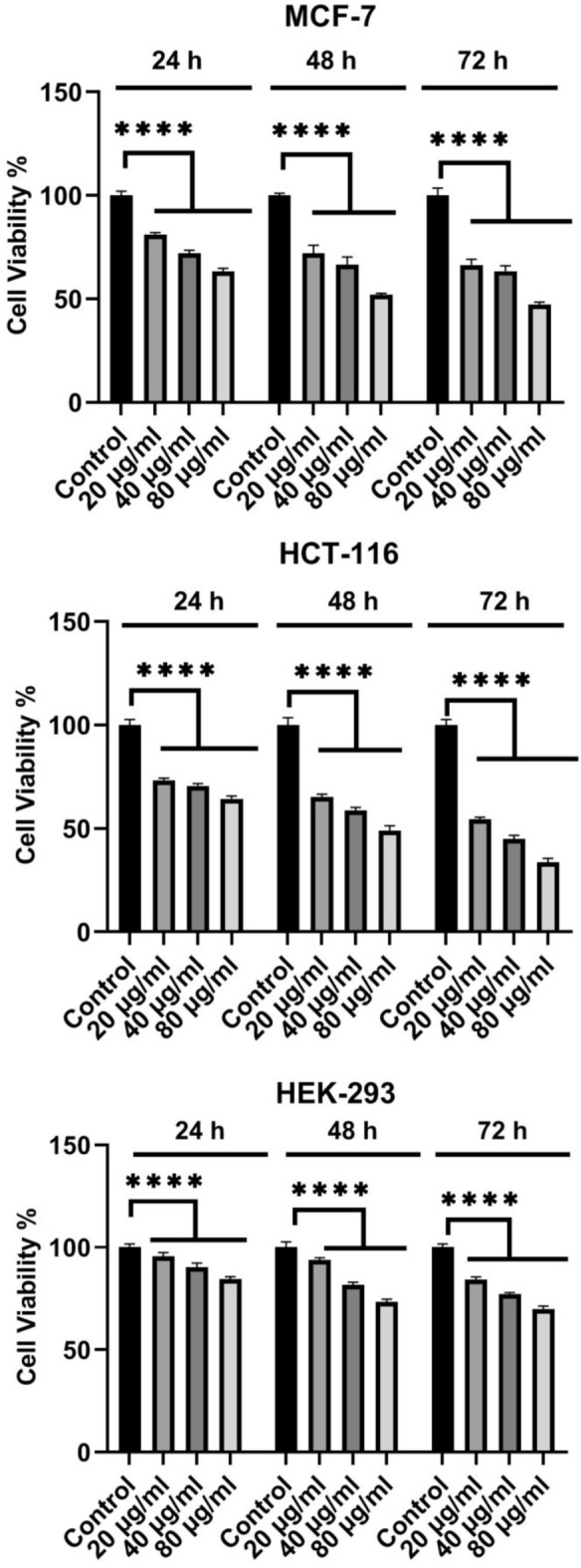
CuO-900 NPscytotoxic effect on MCF-7, HCT-116 and HEK-293 cell lines. CuO-900 suppressed the growth of the cell in all applied concentrations. Control: untreated cells. ****p < 0.0001.

**Fig. 12:**
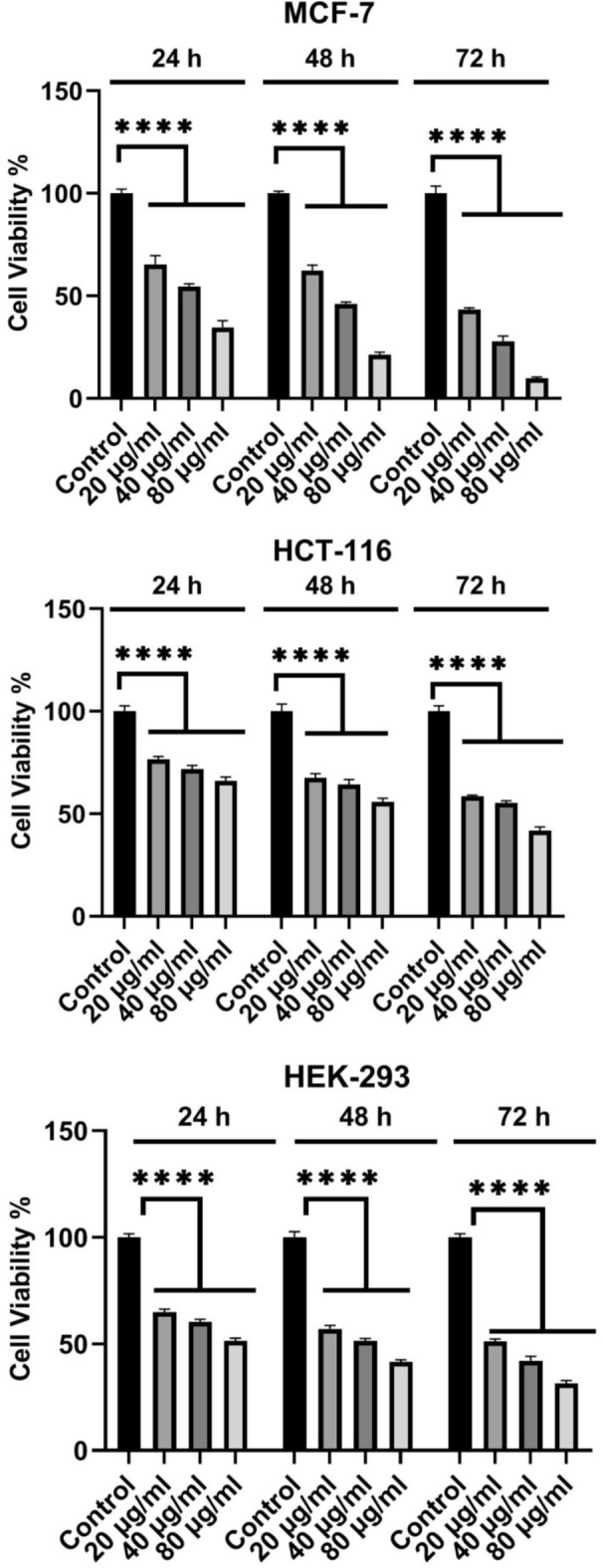
Cytotoxic effects of CuO NPs on MCF-7, HCT-116 and HEK-293 cell lines. CuO-Bulk exerted cytotoxic effect in all concentrations on the cells. Control: untreated cells. ****p < 0.0001.

To determine cytotoxic effects of the synthesized CuO nanoparticles, the MCF-7, HCT-116, and HEK-293 cell lines were treated with different concentrations(20, 40, and 80 µg/ml) of the CuO nanoparticles(CuO-400, CuO-500, CuO-700, and CuO-900) as well as CuO bulk for 72 h (Figs. 8, 9, 10, 11 and 12). Because the CuO nanoparticles exhibited the most cytotoxic effects in 72 h (Figs. 8, 9, 10, 11 and 12), so, the treatment time was chosen to calculate the IC_50_ values (Table 3). After analyzing the MTT data, the nanoparticles IC_50_ values were calculated and summarized in Table 1. As the table showed, the nanoparticles exerted cytotoxic effects on the MCF-7, HCT-116, and HEK-293 cell lines, but the CuO-900 NPs showed lesser cytotoxic effect on HEK-293 cell line(IC_50_ = 330.8 µg/ml, Table 3) compared with the other CuO NPs. Accordingly, the CuO-900 nanoparticles exhibited an appropriate anticancer effect due to their lesser cytotoxic effects on the normal cell line, so, the CuO nanoparticles were chosen for further studies. As Figs. 8, 9, 10, 11 and 12 showed, the cytotoxic effects of all CuO NPs and also the CuO-bulk were in a time and dose-dependent manner. In a more recent work, Dutta et al. reported that biogenic CuO NPs derived from *Erythrina variegate* display cytotoxic effect on HeLa cell line(IC_50_ = 48 µg/ml), but the nanoparticles showed lesser toxic effect on HEK293 cell line^29^. In another work, the CuO nanoparticles synthesized via *Annona muricata* extract reduced breast cancer cells proliferation^10^. Additionaly, in another work, the CuO NPs derived from *Houttuynia cordata* displayed cytotoxic effects on cervical cancer cells^11^. Moreover, Sankar et al*.* have reported that CuO NPs synthesized from *Ficus religiosa* extract reduced A549 cell viability in dose dependent manner^30^. It has been reported that CuO NPs exhibited dose dependent cytotoxic effect on PANC-1 cancer cell line^12^. Therefore, this work results were similar to the previous works. Moreover, our results showed that the anticancer effect of the CuO NPs is correlated to the size of the NPs. The CuO-900 NPs exerted lower cytotoxic effects on HEK-293 cell line rather than other CuO NPs. Because IC_50_ value of the CuO-900 NPs on HUVEC cells was higher than it on MCF-7 and HCT-116 cells(Table 3), therefore, the CuO NPs were chosen as an appropriate anticancer agent to analyze the apoptotic and antioxidant genes expression in MCF-7 and HCT-116 cell lines.

**Table 3:** IC_50_ values(µg/ml) of CuO NPs and CuO-Bulk on HEK-293, HCT-116 and MCF-7 cells.

Cell line	CuO-400	CuO-500	CuO-700	CuO-900	CuO-Bulk
HEK-293	24.59 ± 2.8	30.08 ± 3.7	108.9 ± 9.1	330.8 ± 22.6	22.27 ± 2.4
HCT-116	65.14 ± 5.1	47.98 ± 3.8	128.8 ± 10.4	27.41 ± 3.2	46.56 ± 4.1
MCF-7	84.79 ± 6.3	28.49 ± 3.4	221.7 ± 15.8	75.73 ± 5.7	16.74 ± 1.6

### All of the CuO nanoparticles induced apoptotic cell death

**Fig. 13:**
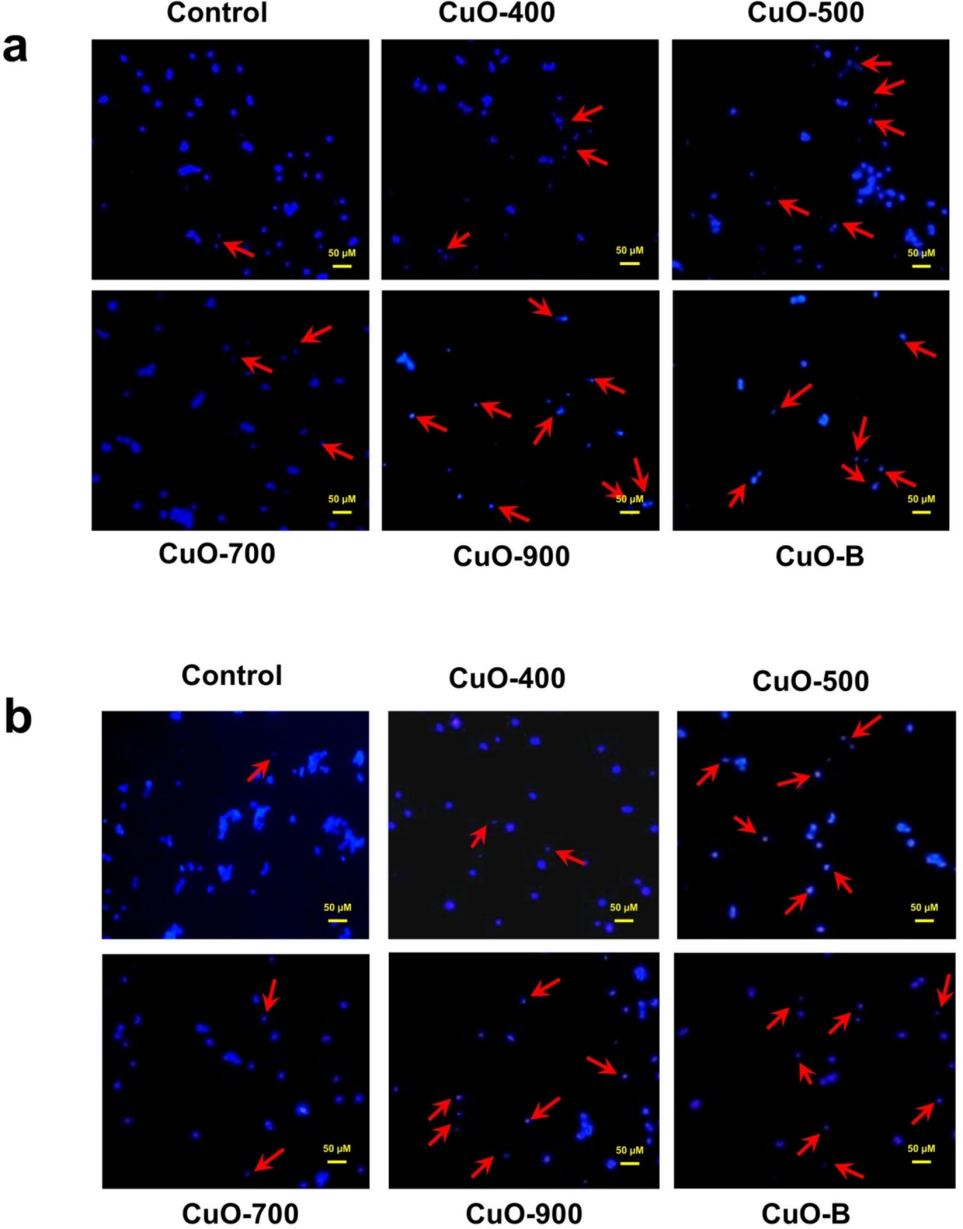
Fluorescence micrographs of Hoechst staining (**a**) HCT-116 and (**b**) MCF-7 cell lines. The apoptotic nuclei are shown by red arrows, in the treated cells the number of the apoptotic nuclei are more than intact nuclei. The most apoptotic nuclei are seen in CuO-900 and CuO-bulk treated cells, respectively. The scale bar is 50 µM. Control: untreated cells, CuO-400: CuO-400 treated cells, CuO-500: CuO-500 treated cells, CuO-700: CuO-700 treated cells, CuO-900: CuO-900 treated cells, CuO-Bulk: CuO-Bulk treated cells.

Apoptosis is a type of cell death that removes unwanted cells without damaging other cells; Therefore, this type of death is an important way for removing cancer cells^31^. Benguigui et al. reported that CuO NPs induce apoptosis in the PANC-1 cell line^12^. In another study, CuO NPs induced apoptosis in HepG2 cells^7^. In apoptotic cells, the cell nucleus undergoes some alterations, including condensation in early apoptosis and fragmentation in late apoptosis^32^. Dutta et al. showed biogenic CuO NPs induce apoptosis in HeLa cells^29^. In this work, the apoptosis inducing effects of the CuO NPs were analysed by Hoechst 33,342 staining to detect the apoptotic cells nuclei. As shown in Fig. 13, in the treated cells, the dense or fragmented nuclei were observed. In the HCT-116 cells, the most apoptotic nuclei were observed in the CuO-900 NPs treated cells, and the least apoptotic nuclei were obtained in CuO-700 treated cells (Fig. 13a). In the MCF-7 cells, the CuO bulk induced the most apoptotic nuclei. Additionally , in the cells, the most and least apoptotic nuclei were seen in CuO-500 and CuO-700 NPs treated cells, respectively (Fig. 13b). The results suggest that the cell viability reduction in the treated cells can be exerted by apoptosis induction. These data confirmed the MTT assay results.

### Effect of CuO-900 nanoparticles on apoptotic genes expression

**Fig. 14:**
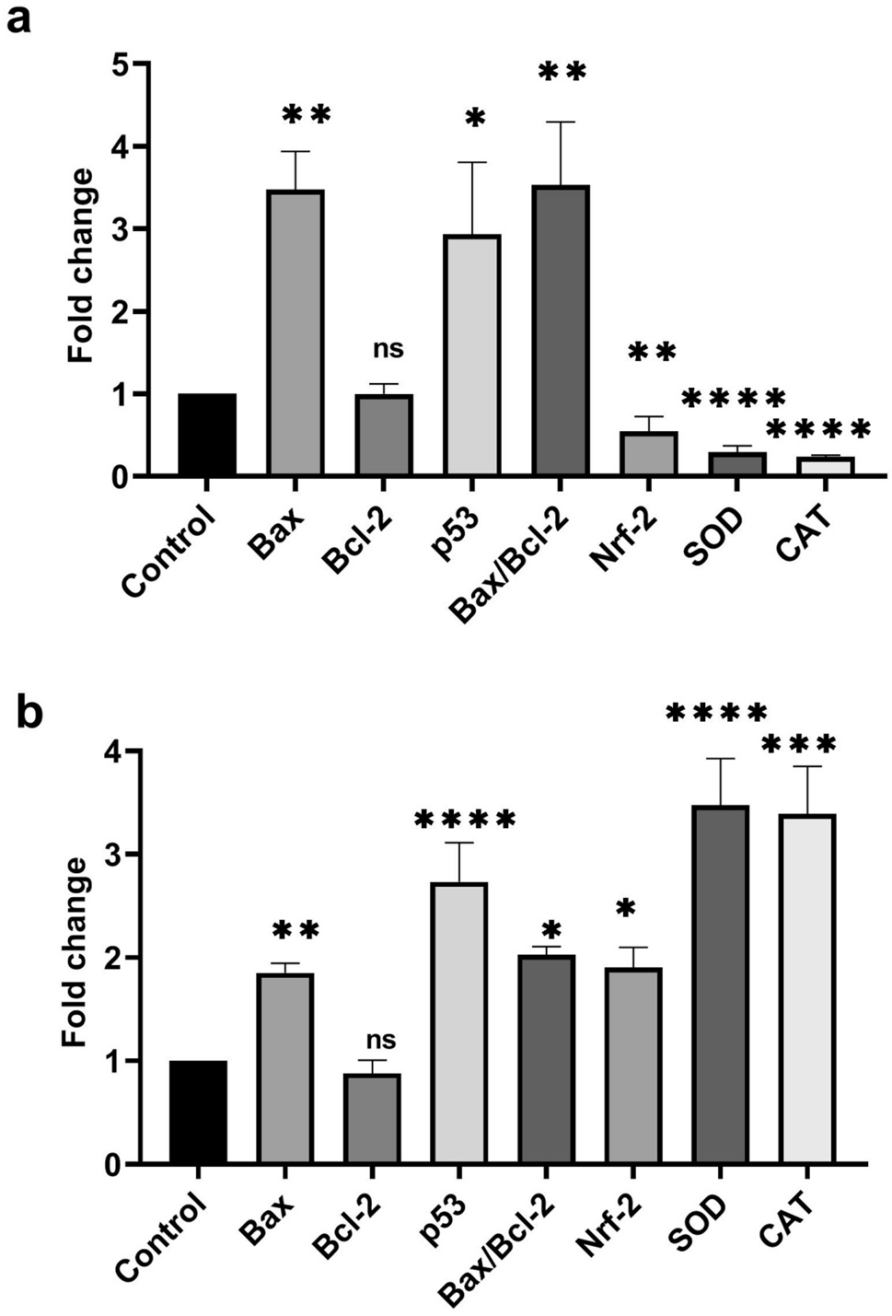
Apoptotic and antioxidants genes expression plots. (**a**) gene expression plot of HCT-116 cells, the expression of Bax and p53 significantly elevated in treated cells, as a while, Nrf-2 gene and other antioxidant genes expression significantly decreased in HCT-116 treated cells. (**b**) gene expression profile in MCF-7 cells, the expression of Bax, and antioxidant genes increased in compared to control cells. *p < 0.05, **p < 0.01, ***p < 0.001, ****p < 0.0001.

To illuminate the role of the CuO-NPs on apoptosis induction, real-time PCR analysis was done. As Fig. 14b showed, in treated MCF-7 cells, the expression levels of p53 and Bax genes were higher than untreated cells (p53:*p*_*control vs treated*_ < 0.0001, Bax: *p*_*control vs treated*_ < 0.05) while the expression level of Bcl-2 was not significantly altered in compared to the untreated cells(*p* = 0.9994). As shown in Fig. 1a, the expression level of p53 and Bax genes was increased in HCT-116 treated cells with compared to untreated cells(p53:*p*_*control vs treated*_ = 0.0016, Bax: *p*_*control vs treated*_ < 0.0001). In another hand, The expression level of Bcl-2 did not exhibit a significant change in treated cells(*p* = 0.999). The Bax/Bcl-2 ratio level in MCF-7 and HCT-116 cells were 1.66 and 3.5, respectively. According to an elevation of the Bax/Bcl-2 ratio level, which is an indicator of apoptosis, the results of the real-time analysis confirmed the hoechst 33,342 staining results that indicating apoptosis occurence in CuO-900 NPs treated MCF-7 and HCT-116 cell lines. The results are similar to a recent work’s results, which reported CuO NPs derived from *Bacillus Coagulus* elevate the Bax/Bcl2-2 level in MCF-7 and SKBR3 cell lines^33^. It was reported that DNA damage and reactive oxygen species increase the p53 gene expression^34^. Therefore, CuO-900 NPs may increase the p53 gene expression by DNA damage or ROS production, which leads to apoptosis induction.

### CuO-900 NPs exhibit different effect on Nrf-2 gene expression in the MCF-7 and HCT-116 cell lines

The antioxidant activity of the CuO NPs was measured by the DPPH test. As Fig. 7 showed, the CuO NPs did not display antioxidant activity. Despite , in previous works, the green synthesized CuO NPs exhibited antioxidant activity^35–37^. According to the application of high temperatures in the synthesis procedure of the CuO NPs, it can be suggested that the high temperatures degrade the organic compounds which cause antioxidant activity. To investigate the effect of CuO-900 NPs on the antioxidant genes expression profile, real-time analysis was employed. Nrf-2(nuclear factor erythroid 2-related factor-2) is a transcription factor which plays a pivotal role in the redox state of cells by overexpressing of own gene expression as well as other redox genes expression, such as superoxide dismutase(SOD) and catalase(CAT) enzyme genes^38–40^. Therefore, Nrf-2 overexpression reduces the oxidative stress in cells^41^. Based on the role of the Nrf-2 , superoxide dismutase and catalase in the redox state of the cells, their gene expression level were chosen to investigate the redox genes expression analysis in the CuO-900 NPs treated cells. Tabatha et al. showed that CuO NPs activate the Nrf-2 pathway in the JB6 cells^42^. As shown in Fig. 14b, the Nrf-2 and SOD and CAT genes expression level was increased in MCF-7 treated cells (1.9, 3.4, and 3.3 times, respectively). The effect may be caused by CuO-900 NPs mediated ROS production that activates the Nrf-2 signaling pathway. Reversely, in the HCT-116 treated cells (Fig. 14a), expression level of Nrf-2, SOD, and CAT genes was decreased in compared to untreated cells (0.54, 0.29, and 0.23, respectively). Chen et al. reported that the Nrf-2 signaling pathway is suppressed by a high level of p53 protein^43^. Therefore, the suppressive effect of the CuO-900 on Nrf-2 expression may be a result of the NPs caused p53 gene overexpression in HCT-116 line. According to the results, based on the higher IC_50_ value of CuO-900 on MCF-7 cells in compared to HCT-116 cells, the different effect may be correlated to the MCF-7 cells’ lower sensitivity to the CuO NPs rather than HCT-116 cell line.

## Conclusion

The CuO NPs were synthesized using a Walnut shell in four different temperatures for first time. The size of CuO NPs was increased by raising the calcination temperature. All the CuO NPs showed cytotoxic effect on MCF-7, HCT-116 and HEK-293 cell lines, but the CuO-900 NPs exhibited lesser cytotoxic effect on normal cell line with compared to other CuO NPs. The CuO NPs did not exhibit antioxidant activity, on the another hand, the CuO-900 NPs showed opposite effects on the expression of the Nrf-2 gene expression in the HCT-116 and MCF-7 cell lines. Furthermore, the CuO-900 NPs increased the expression of Bax and p53 genes in the HCT-116 and MCF-7 cell lines, indicating apoptosis occurence in the cell lines. According to the bigger size of the CuO-900 NPs compared to other CuO NPs, it can be suggested that CuO NPs’ anticancer effect is size dependent. Consequently, the Cuo-900 NPs may be a promising anticancer agent to suppress breast and colon cancers.

## Data Availability

The datasets used and/or analysed during the current study available from the corresponding author on reasonable request.
